# Training family physicians: A qualitative exploration of experiences of registrars in a family medicine training programme in Cape Town, South Africa

**DOI:** 10.4102/safp.v62i1.5023

**Published:** 2020-02-25

**Authors:** Tasleem Ras, Beverley Schweitzer, Graham Bresick, Derek Hellenberg

**Affiliations:** 1 Department of Public Health and Family Medicine, Faculty of Health Sciences, University of Cape Town, Cape Town, South Africa

**Keywords:** qualitative programme evaluation, learning environment, professional identity formation, family medicine, primary care

## Abstract

**Background:**

The MMed in Family Medicine is a professional Master’s qualification spanning 4 years of training. The outcomes were predetermined by national consensus. While these outcomes are measured in the form of a national exit examination, there has been no exploration of the experiences of registrars (residents) in this relatively new programme. To evaluate the experiences of registrars in one of the nine training programmes in South Africa and to identify areas for improvement.

**Methods:**

This study used purposive sampling to recruit registrar (*n* = 9) and supervisor (*n* = 8) participants into respective groups. Data were collected via semi-structured interviews and analysed thematically, and consensus was built using the nominal group technique.

**Results:**

Supervisors identified the strengths and weaknesses of the programme which will impact on further strategic planning. Data from registrar interviews yielded two themes: affirmation, referring to the positive social engagement and facilitation of professional identity formation; and frustrations, referring to structural aspects of the programme which hindered academic progress.

**Conclusion:**

Qualitative programme evaluation is a useful tool in understanding the learning environment. The student perspective helped to identify the unintended consequences of the programme. It was also shown that the nominal group consensus building technique worked well in a resource-constrained environment.

## Introduction

The registrar programme in Family Medicine (FM) has been running at the University of Cape Town (UCT) since 2008. To date, 14 family physicians (FPs) trained in this programme have successfully completed all requirements to register on the specialist registry of the Health Professions Council of South Africa (HPCSA), and 16 registrars are currently in various stages of the 4-year programme. The curriculum has been nationally agreed upon, guided initially by the Family Medicine Education Consortium (FaMEC^1^), constituted by representatives from all the FM departments in South Africa (SA). The Consortium has since been replaced by the Education and Training Committee (ETC) of the SA Academy of Family Physicians (SAAFP). Each university has implemented the 4-year training programme according to available resources and interests. The first batch of graduates of the UCT programme have entered the workforce and assumed positions as FM consultants, and would have well-developed ideas around their experiences of their training programme.

As part of the ongoing process of reflection aimed at constantly improving the training programme at UCT, this qualitative study explored the perceptions of supervisors and the experiences of registrars. This was deemed necessary to gain deeper insights into the learning environment (i.e. the physical or virtual setting in which learning takes place), the factors affecting academic performance and professional identity formation (PIF [The process of developing an identity as a medical professional based on experiencing the formal and informal curriculum])^2^ of these new FPs and to provide useful data for critical reflection on the pedagogical approach utilised.

## What is already known?

Understanding the student experience is an important factor in understanding academic performance.^3^ Factors impacting on the overall performance and educational experience go beyond the formal curriculum and its learning objectives, as shown by various South African studies^3,4^ wherein students cited the informal interactions and relationship-building between each other and lecturers as significant factors in their educational experience. Wayne and colleagues showed a statistically significant correlation between student perceptions of the learning environment and actual test scores performance in a United States medical school.^5^ Similar to Bannister and colleagues, we understand the ‘learning environment’ to be a combination of physical, emotional, psychological, organisational and social experiences that students have while engaging in their studies.^6^

The training of FPs in the South African context is rooted in a deep understanding of the socio-cultural demands placed on the health system.^7^ Although relatively new in medical education,^8^ the development of postgraduate FM training programmes has followed a thorough process that has resulted in a consensus-driven, broadly accepted curriculum.^1^ In addition, the training programme has been framed by a learning portfolio that has been generally well received by faculty and students across SA.^9,10^ The desired outcomes of the FM curriculum have been captured within five Unit Standards describing the competencies that FPs are required to master to enable them to operate in the SA context.^1^ In addition, the roles that FPs play in the health system have been categorised into six roles, indicating the broad scope of practice that the training programme must cover.^11^ To date, no studies have documented the overall experiences of registrars in any of the FM registrar programmes in SA.

A qualitative method of determining perspective among participant experiences is regarded as an important component in gaining a deeper understanding of the educational experience.^12^ This method allows the researcher to probe issues that are not normally accessible using quantitative methods of data collection in an in-depth manner.

This study qualitatively explored the perceptions of educators and the experiences of registrars in one of the nine^8^ postgraduate FM registrar programmes in SA, with the aim of understanding factors that are not easily identified quantitatively, which can enhance or inhibit learning and professional development.

## Methods

This was a descriptive study in which qualitative methods (including data collection and analysis) were employed.

The setting of this study was an urban-based postgraduate training programme. At the UCT, the clinical training platform consists of generalist (FP) run primary care clinics and specialist run departments in district hospitals. In addition, the Division of Family Medicine convenes weekly lectures on various topics. Registrars are rotated through various clinical attachments every 3 months, with each placement having a well-defined training need that is aligned with the overall objectives of the programme. Typically, the FP supervisors are active as researchers and educators in the Division of Family Medicine, while the specialist supervisors have minimal interaction with the department beyond the clinical supervision of registrars.

Two groups were identified: educators and students.

Two educator groups were purposively selected from all educators involved in teaching registrars in the UCT FM programme. The first group consisted of supervisors working in the District Hospital, and the second comprised those working in primary care clinics. It was expected that these two groups may have had different experiences of the training programme given their differing disciplines and contexts of work, hence the need to meet with them separately. Educators were invited to participate in a focus group (FG) discussion with their peers. The hospital-based FG met at the hospitals to minimise time away from the clinical site. Although efforts were made to include all educators, clinical service demands meant that not all of them could attend the FG. A total of eight educators participated in this round of data collection. The nominal group technique (NGT) was used to obtain group consensus on key features of the programme, guided by GB, a member of the research team, who has experience with this technique. Two NGT sessions were held: One with hospital-based (*n* = 4), and one with primary care–based clinical supervisors (*n* = 4). Two key questions were asked from both these groups: ‘what do you perceive as strengths of the programme?’ and ‘how can the programme be strengthened?’ The NGT process generated a list of ‘strengths’ and ‘what could be better’ in each group. Participants ranked each of these items (responses) in order of perceived importance to obtain consensus on the top-ranked items in response to the questions. These data were used in a strategic planning session that was not part of this project.

The members of the student group were purposively recruited from among previous students and graduates who had spent more than 2 years in the programme. The reason for this inclusion criterion was to better understand student experiences after undergoing the training for a significant amount of time, which we deemed to be 2 years. Students who were enrolled in their third or fourth year at the time of the study were excluded, as it was anticipated that their vulnerability as students would have influenced their responses. A total of 13 participants were eligible: 12 had completed the 4-year clinical training, and one had left the programme in the third year for personal reasons; all were invited to participate in a semi-structured interview. Of these, nine were available to be interviewed. Reasons for non-participation included being too busy^12^; no response to email or telephonic messages.^1^ A summary of the demographic details of the participants is found in Table 1. The interviews were conducted by T.R., the main author, at a venue and mode based on the participant’s choice.

**TABLE 1:** A summary of student participants’ demographics.

Participant	Age (years)	Gender	Nationality	Duration on the programme (years)	Time since leaving the programme (years)
1	36	M	SA	4	1.5
2	35	M	SA	4	0.5
3	37	F	SA	4	0.5
4	39	M	Nigeria	4	1.5
5	34	F	SA	4	1.5
6	41	F	SA	4	2.5
7	33	F	SA	4	0.5
8	34	M	SA	4	0.5
9	36	F	SA	4	2.5

M, male; F, female; SA, South African.

The interview guide was developed by the research team with the intention of exploring participant-identified experiences that they deemed important. In addition, questions 9 and 10 were inserted following specific feedback from students when conducting routine course evaluations. This guide was reviewed by an expert in the field with experience in postgraduate FM teaching and qualitative research. After incorporating the recommendations of this reviewer, a pilot interview was conducted to further validate the tool. Participants were asked to consider and comment on questions in Box 1.

BOX 1 The semi-structured student interview guide.

1. What are your general impressions of the programme as you experienced it?
2. Were there particular strengths that you experienced?
3. Did you have any particularly good experiences?
4. Did they leave a lasting impression on your professional development?
5. How can the programme be strengthened?
6. Did you have any particularly bad experiences?
7. Did they leave a lasting impression on your professional development?
8. How did you find the research?
9. How was the busyness of the academic components/coursework?
10. Was there anything from outside the programme that impacted on your professional development? (Not wanting to probe too personally, but in an attempt to understand context)

Eight interviews were conducted face to face; the ninth interview was conducted via Skype. Interviews lasted for an average of 46 min and 17 seconds (range: 30’54” – 59’08”) and were digitally audio recorded. They were subsequently anonymised and transcribed into Microsoft Word documents before being uploaded to Nvivo v 11.0^13^ and analysed thematically.

The main author (T.R.) conducted the data analysis. This involved reading and re-reading of transcripts immediately after transcription. Data extracted from the transcripts were coded. These codes were categorised and organised into themes. No new themes were identified after the analysis of the fifth interview but we continued interviewing and analysing the data of all nine participants to ensure that saturation was reached. The themes were presented to the student participants by email. Although only two out of the nine responded to the call for feedback, no modifications were proposed by the participants. The final themes are discussed further.

### Ethical considerations

Approval for this study was obtained from the Human Research Ethics Committee of the Faculty of Health Sciences, University of Cape Town (Reg. no.: 866/2015). The study complied with all the conditions stipulated by the Declaration of Helsinki on research involving human subjects.

## Results

### Educator or supervisor group

In response to the questions ‘what are your perceptions of the strengths of the programme?’ and ‘how can the programme be strengthened?’, the educator-participant NGT groups from the respective clinical sites generated a range of responses (Table 2).

**TABLE 2:** Supervisor-participant focus group identification and ranking of top five responses to the key questions.

PHC supervisors	Hospital supervisors
***What are the strengths of the family medicine registrar training programme?***	
Workplace-based learning opportunities Programme structure enables perspective and consolidates learning Curriculum facilitates learning and growth Good role-modelling Win-win (reciprocity): Benefits to students, health facility and supervisor	Strong learning environment Burden of disease – good case mix and load Good team ethos at work Diverse teaching team Family physician role model in a multi-disciplinary team/curriculum is good/clinical governance component
***How can the programme be strengthened?***	
Strengthen the teaching programme Clarify and standardise roles and expectations Strengthen the curriculum Improved selection process Appropriate role-modelling Guidance in career development	Registrar selection Strengthen the curriculum Basic clinical skills More time in specific disciplines More FP role models on platform

PHC, primary health care; FP, family physician.

In relation to the programme’s strengths, there was an agreement between the two groups that students were afforded sufficient workplace-based opportunities for learning and that good role models were to be found within the programme. Both groups also agreed that improved registrar selection, curriculum strengthening and more FP role models on the training platform would strengthen the programme. In clarifying ‘curriculum strengthening’, educators indicated that they were referring to the predetermined learning objectives defined in the logbook (the section of the portfolio defining which clinical skills need to be acquired) and clearly identifying the roles of supervisors and students in achieving these objectives.

### Student cohort

Analysis of the student interview data generated two broad themes: affirmation and frustration. Affirmation refers to the experiences that students had which affirmed their technical learning and PIF. The subthemes that formed this theme were identified as PIF and positive social engagement.

#### Professional identity formation

Several participants reported that they developed a sense of their professional identity as FPs by drawing on the skills that their training provided for them in diverse contexts. This was particularly apparent when problem-solving in multi-disciplinary teams where FM registrars were asked to do so in lieu of their enhanced communication skills and training within the biopsychosocial paradigm. As one of the participants reported:

‘[…*I*]t happened many times. If someone … if someone would be dying, they would call me to speak to the family. Once there was a patient who refused to have an operation and then they called me to come and speak to the patient.’ (T2, Male, 35 years)

This professional identity was also affirmed when registrars became aware of competencies they had achieved when getting feedback from various people who commented on their skill set, whether clinical or interpersonal. This provided a sense of self-efficacy, reinforcing a sense of (self) belief in the discipline of FM. In commenting on her interaction with a group of students who observed her dealing with a difficult clinical problem, one of the participants reported:

‘… I’ve had a bunch of students comment on it when I didn’t even realise I was doing it. So, it’s stuff that was being taught in the programme that’s become a part of my professional philosophy without realising, which is good I suppose.’ (T8, Female, 36 years)

Participants also reported that the presence of role models was an important contributing factor to their learning. The key characteristics cited in a role model were compassionate interaction with patients, excellent organisational skills and high levels of professionalism. Interestingly, none of the participants mentioned proficiency in technical skills, for example, surgical or emergency procedures – as core skills that they looked for in potential role models.

#### Positive social engagement

Participants were unanimous in stating that they felt supported throughout their training and mentioned this positive social engagement with colleagues and faculty as a key enhancing factor in their educational experience. This became particularly important when they were faced with difficulties. After relating a particularly painful experience wherein his ongoing participation in the training programme came under threat, this support from faculty was made explicit by one of the participants:

‘… I enjoyed the support of everybody involved … They gave us chances to chat (name deleted) was always very supportive, (name deleted) as well. They started with us and then as time went on, they were always there to answer questions. Even (name deleted) would be able to address us, we had some queries upfront. And he was always there, always open, always available, so there was always good support.’ (T1, Male, 36 years)

In addition to the faculty support, all participants identified the regular contact sessions with their peers as a major coping mechanism. In the context of their clinical work being conducted in various health facilities and departments, the weekly contact sessions (registrars meet on campus once a week to review theoretical aspects of the curriculum) allowed them to reconnect with peers and reflect on their practices. The following statements summarise much of what was reported by all participants:

‘So, we had that weekly contact, not just by having a contact session with a family physician, but by also seeing your peers, your colleagues and also learning from each other.’ (T2, Male, 35 years)

‘[*T*]here’s a platform for you to ask things and you know the people, you have more, you feel free to say, to ask things. Say, when something bothers you, so to me, the contact sessions are good. And, that also strengthens what I was saying, that “family” feel. That we’re all registrars, we still know each other, we had a group, like, a WhatsApp group where we all supported each other and asked questions.’ (T7, Male, 33 years)

#### Frustrations

Frustrations expressed by the participants predominantly referred to some aspects of the organisation of the formal academic activities such as difficulties with research and with implementing some of the theoretical aspects of FM in very busy clinical settings.

In a training programme with a heavy clinical and academic workload, the perceived lack of organisation in formal learning activities was mentioned by most participants as follows:

‘[…]Sometimes we felt that the organization was a bit haphazard. Like your…like some sessions were cancelled or in the afternoon, someone wouldn’t pitch up.’ (T4, Male, 39 years)

‘[…*S*]o, it’s just, kind of, those logistical things but I just think that that has a major impact on how people perceive the course even if the clinical content is great and the exposures great, if it feels haphazard and disorganised, it’s going to give people a bad impression.’ (T8, Female, 36 years)

While the research component is a prerequisite to earning the degree, participants felt that not enough support was forthcoming, particularly in statistical analysis of their research data. For most of them, research was a completely new endeavour, and they often felt lost in this process. The following quote demonstrates the struggle of overcoming this challenge while at the same time dealing with all the other responsibilities in the programme:

‘[…*E*]ven though you are postgraduate, you have so many responsibilities, overtime, you’ve got your portfolio, you have to study, you’ve got to work, it’s the normal working day and all these things, and you don’t always know … And, like, stats, but I think that’s a problem all over.’ (T7, Male, 33 years)

While reflecting on their experiences in the programme, participants reported two areas of uncertainty: the lack of posts for qualified FP’s in Cape Town and the huge demands on clinical services which limited the practice of the biopsychosocial model of care. Both these issues were obviously significant to participants. However, it is not a direct reflection of their experiences in the programme. Lack of permanent consultant positions and the heavy clinical workload are situations to be addressed by the public health sector and are beyond the scope of this study.

## Discussion

Performing a qualitative programme evaluation, which is defined as a qualitative assessment of various stakeholders’ experience of a programme,^2^ revealed insights into the academic programme that were not forthcoming in our previous quantitative evaluations. While the standard quantitative evaluations (unpublished routinely collected programme data) raised issues mainly related to programme management and student performance, and are driven by institutional imperatives, the qualitative approach was able to identify experiential aspects from the perspectives of students and educators, providing a more grounded understanding of their experiences. This finding is in agreement with reports from other programmes in the same university^3^ and with the North American assertion that the learning environment impacts personal and professional development.^6^ We now have a better understanding of how structural aspects of the training programme influence the learning environment in beneficial and non-beneficial ways.

The supportive elements of structured academic time away from the clinical environment, opportunities for socialising between students, the surprising finding that registrars value role models more for their professional attitudes than technical expertise and the opportunity for registrars themselves to be recognised as role models to students offer opportunities for further enhancing aspects of the ‘hidden curriculum’ that were deemed beyond our reach.

We were intrigued by the themes relating to PIF as FPs: registrars organising themselves into a ‘community of practice’^14^; supportive faculty–student relationships and positive affirmation based on value in the multi-disciplinary teams all contributed to this process of PIF. This stage of PIF could be categorised as Kegan stage 4 (institutional), where the values of the institution are internalised, while maintaining a keen sense of self and value in relation to others.^15^ In our setting, the ‘institutional values’ referred to are the values taught as part of the FM postgraduate programme relating to the doctor–patient encounter and self-awareness. Students were engaging critically with their working environment and clearly identifying areas of divergence or convergence with their own values. This surprising and encouraging finding could best be characterised as an unexpected effect of the training programme.

The major role that individual power (agency) played in how registrars negotiated their way through complex clinical systems also deserves mention as this exemplified the notion that individual learning and socio-cultural context are ontologically separate, while at the same time being highly interdependent.^16^ While the programme is framed within a self-directed learning ethos implying personal responsibility, our findings indicated that as registrars rotated from one clinical team to the next, it was this sense of agency as developing FPs focussed on optimising patient-centred care that allowed them to interact critically with the learning environment. Whether this was because of a pre-existing personality trait before entering the programme, or one which was enhanced by the programme is beyond the scope of this study, but which certainly warrants further attention.

Our experience in using the NGT to build consensus from the supervisor group has some support in the literature.^17^ With limited time resources, we were able to develop consensus on the perceived challenges and weaknesses within the programme. The immediacy of the consensus allowed us to verify with the participants whether the findings as reported here were accurate and trustworthy.

In response to the concerns regarding slow progress with the research component of the degree, we have now formalised timeframes for completing certain sections of the project and strengthened technical support in areas of study design, academic writing and data analysis.

### Limitations

A key limitation to this study is that it did not include registrars who did not complete the 4-year programme. One of the main reasons for this is that our inclusion criteria stipulated that students should have been on the programme for a minimum of 2 years. All, except one, of the registrars who left the programme did so during the first and second years, automatically excluding them from this study. The one registrar who left in the third year was not available for an interview.

A second limitation is one generally applicable to qualitative studies in that the findings cannot be generalised to other FM programmes because of the specific nature of student experiences in this environment.

## Conclusion

Using qualitative methodology, we aimed to explore how structural aspects of our postgraduate training programme influenced learning and identity formation. This offers a useful approach to programme evaluation, offering deeper and unique insights into often unintended consequences of teaching programmes. Future studies should evaluate identity construction from the perspectives of personal and professional values and narratives.
